# Understanding students’ problem-solving patterns: Evidence from an allotted response time in a PISA 2012 item

**DOI:** 10.3389/fpsyg.2022.1050435

**Published:** 2023-01-04

**Authors:** Hyun-Jeong Park, Dayeon Lee, Hyemin Park

**Affiliations:** Department of Education, Seoul National University, Seoul, Republic of Korea

**Keywords:** process data, response time analysis, process map, learning process, problem-solving patterns, PISA 2012

## Abstract

Understanding students’ learning characteristics is central to successfully designing student-centered learning. Particularly in the problem-solving area, it is vital to know that students can possess their styles to solve problems, which should be considered central to addressing adaptive learning. To date, analyzing students’ learning characteristics has been mainly based on their final answers. However, there is a limit to understanding the thinking process of students with the correct answer, because their responses are, *de facto*, singular and identical. With this background, we propose an approach for investigating students’ cognitive behavior in problem-solving using response time in the process data. In this paper, we analyzed an item in Programme for International Student Assessment 2012 Creative Problem Solving (CP038q1). We analyzed log data from the PISA CPS item *Ticket* encompassing 30,453 students (7,196 students with the correct answer and 23,257 students with incorrect answers) from 42 countries. We found that students with the correct answer are categorized into four clusters, and the problem-solving patterns of each cluster are distinguishable. We also showed the internal validity of this approach by confirming that students with incorrect answers can also be similarly classified. Our results indicate that allotted response time in an item can shed light on several distinguished problem-solving patterns, which implies that adaptive learning and feedback are vital for them.

## Introduction

One of the important purposes of educational evaluation is to establish effective teaching strategies and provide productive feedback to students based on reliable and valid estimates of students’ abilities, thereby improving the quality of subsequent education (Baek, 2019). For this purpose, educational assessments are carried out in various ways as technology is increasingly highly developed. Particularly, in computer-based tests, every mouse click and keystroke during the problem-solving process is recorded in log files with timestamps, and collecting these data has facilitated novel forms of assessment (Wang, 2021). In other words, by using computers in the educational field, it becomes possible to record the problem-solving process of taking tests and determine more about students’ particular problem-solving patterns and deployed strategies (Shin, 2021). Process data have enriched educational assessment and evaluation beyond simply providing information on what are correct or incorrect answers. For example, He et al. (2019) analyzed students’ problem-solving proficiency using process data from the Programme for the International Assessment of Adult Competencies and found that problem-solving patterns and strategies are closely related to problem-solving proficiency. This study showcases the potential that log data analyses have for utilization in terms of learning analytics through the visualization of problem-solving patterns using log data. Therefore, using time-relative variables derived from log data, our study seeks to identify different problem-solving patterns.

### Previous studies on problem-solving strategies using PISA 2012 process data

Following significant advances in educational assessment, PISA has implemented computer-based assessment (CBA) since 2006. In 2012, three areas of “digital reading,” “mathematics,” and “problem-solving” were designed as within the purview of CBA (OECD, 2014a). Problem-solving was one of the core components of PISA 2012, in that assessment using computers is suitable for interactive items, where some exploration is required to uncover undisclosed information (Ramalingam et al., 2014; OECD, 2014a). PISA 2012 defined complex problem-solving skill as “an individual’s capacity to engage in cognitive processing to understand and resolve problem situations where a method of solution is not immediately obvious. It includes the willingness to engage with such situations to achieve one’s potential as a constructive and reflective citizen” (OECD, 2014a, p. 30). After PISA 2012 released the process data, it created an opportunity to expand the depth of assessment, and various studies on students’ problem-solving processes and strategies were conducted. For example, Greiff et al. (2015) defined vary-one-thing-at-a-time (VOTAT) as an important problem-solving strategy that can be applied to PISA 2012 *Climate Control* items, which require students to find the function of the three buttons on the climate control, humidity and temperature, and identified the relationship between VOTAT usage and item correctness. They then discussed the use of log data in educational assessment. Also, some studies proposed new methods of utilizing process data. For instance, Han et al. (2019) adapted n-grams to generate action sequence features based on VOTAT. Then they selected features using random forest and backward elimination, showing n-grams can be translated into mini-sequences along with their frequencies. Ren et al. (2019) showed the usability of students’ goal pursuit in an item context to analyze log data. They defined three possible problem-solving goals in a PISA 2012 *Ticket* item and clustered students based on their goal pursuit to identify the relationship with CPS proficiency. Meanwhile, other studies showed that process data can reveal the differences in problem-solving ability between boys and girls (Wüstenberg et al., 2014), students with and without a migration background (Sonnleitner et al., 2014), and interaction effects of gender and migration background (Eichmann et al., 2020).

### Learning analytics

The problem-solving pattern can be defined as behavioral characteristics captured in the process of problem-solving that reflects strategies, time, and orders of problem-solving. Each learner has a different problem-solving pattern, and it is necessary to deploy suitable teaching strategies to learners’ characteristics to facilitate effective learning (Beck, 2001). Kolb and Kolb (2005) also suggested a few types of learning styles and emphasized the necessity of providing students with a learning experience that is appropriate to their characteristics. Indeed, learning analytics can be helpful for teachers and students in exploring unobserved patterns and underlying information in learning processes (Agudo-Peregrina et al., 2014; Yin et al., 2019). Problem-solving pattern analysis is a significant issue in learning analysis. Moreover, in paper-based assessment, it is almost impossible to obtain detailed information about students’ problem-solving processes or patterns, so there is a *de facto* limit to understanding their problem-solving characteristics. To overcome this, many studies have approached the students’ problem-solving strategies through self-report questionnaires (e.g., Pereira-Laird and Deane, 1997; Danek et al., 2014). However, the results of self-reporting questionnaires can be biased because they can reflect “what students think of themselves” and not “what they really are.” Recently, with the introduction of CBA, it has become possible to access and analyze students’ problem-solving processes and strategies directly through the process data (e.g., Greiff et al., 2015; Han et al., 2019; Ren et al., 2019). Many studies that analyzed the students’ problem-solving using the process data identified students’ specific problem-solving strategies that were applied to specific items. However, studies on comprehensive patterns and strategies are somewhat scant.

### Response time

The concept of response time is drawing the most attention within the wider process data paradigm. By analyzing response time, not only can we confirm whether the test was conducted properly (e.g., Yamamoto and Lennon, 2018) but also infer how much the students were engaged in the test (e.g., Goldhammer et al., 2016). Also, the response time provides useful information for an in-depth understanding of students’ various ways to approach and solve problems (He et al., 2018; Shin, 2021).

In general, response time can be examined from various aspects. First, different explanations are possible from the perspective of speed. For example, a long response time by a student can be interpreted positively in that they addressed the item carefully, whereas it can be interpreted negatively in exams where speed is an important factor. Many studies have analyzed the relationships between response speed and students’ accuracy or ability (Swanson et al., 2001; Lee, 2007; Goldhammer et al., 2015; Shin, 2021; Ulitzsch et al., 2021).

Second, response time can be analyzed at either the test-level or item-level. Particularly, in large-scale, low-stakes assessments like PISA, students can determine their problem-solving speed and spend a given amount of time on each item as they wish. In this case, the response time can be a real indicator of students’ problem-solving behavior. Studies that consider response time at the test-level, which analyzes how much time is spent on each item in an entire test, can show how well students behaved adaptively according to the cognitive loading each item requires. This is also called the “time-on-task effect” and many studies have been carried out to identify the relationship between both item difficulty and students’ learning characteristics (e.g., Goldhammer et al., 2015; Naumann and Goldhammer, 2017; Naumann, 2019).

Conversely, if the response time is analyzed at the item-level, by subdividing the total response time into specific steps, we can identify students’ cognitive processes and strategies to address the item (e.g., Hahnel et al., 2022). In particular, if an item consists of several decision-making steps, like those items in the PISA 2012 CPS task, the time-related variables for each step of addressing the item can be identified. In this way, it is possible to recognize which step the student spent a long time on, or focused on. Also, the student’s problem-solving behavior or pattern can be inferred from the configuration of time spent on the item (Whimbey and Lochhead, 1991; Zoanetti, 2010; Eichmann et al., 2019). For example, even if the total amount of time that students spent on an item are the same, the student who spends a long time reading around questions of items and then shaping a problem-solving strategy, and the student who spends most time clicking and exploring the problem, has different problem-solving strategies (Goldhammer et al., 2014). Thus, item-level analysis can reveal the students’ particular cognitive processes and patterns.

In short, studies related to response time have analyzed response speed or the time allotted to the either test-level (e.g., Engelhardt and Goldhammer, 2019) or item-level (e.g., Ren et al., 2019; Hahnel et al., 2022). Still, with test-level analysis, it is difficult to identify the precise step in which students spend a lot of time on an item. The whole problem-solving process can be broken down into detailed steps, and how much time each step takes can be treated as a piece of evidence that shows the student’s problem-solving pattern and strategy. Therefore, analyzing the time allotted to each step in an item can be a credible way to check how the students addressed the problem or what problem-solving pattern they generally possess.

### The purpose of this study and research questions

The purpose of this paper is to identify and understand students’ problem-solving processes. To this end, we analyzed an item in PISA 2012 CPS (CP038q1) and determined the various problem-solving patterns among students who addressed this item correctly. We propose a method to identify problem-solving patterns using response time as the key variable. This study can evidence the necessity of devising effective teaching methods according to the cognitive styles of students and provide productive feedback not only for students with incorrect answers but also for students with the correct answer. Furthermore, based on the findings, we would like to suggest the importance of understanding students with various problem-solving patterns and implementing adaptive teaching methods adapted to the learning characteristics of students.

Based on the objectives, the research questions are as follows:

- RQ1. What time-related variables predict the problem-solving ability of students who provide the correct answer?
- RQ2. What are the differences in the problem-solving patterns of students clustered with selected time-relative variables?
- RQ3. How does the problem-solving pattern of students with incorrect answers differ from those of students with the correct answer?

## Materials and methods

### Procedure

We analyzed log data from the PISA CPS item *Ticket* (CP038q1) encompassing 30,453 15-year-old students (7,196 students with the correct answer and 23,257 students with incorrect answers, 15,152 boys and 15,301 girls) from 42 countries. For the students, we analyzed how much time is spent on each problem-solving step, and how the students are then clustered based on the time spent. Also, we present the differences between the clusters using descriptive statistics and process maps. A process map is a tool that has been mostly used in the business field to date (van der Aalst, 2016). We take advantage of it here to visualize how students in each cluster addressed the item and precisely how much time they spent on each action. Specifically, as in the PISA 2012 CPS, where the items consist of several stages of initial exploration and final decision-making, various paths can be explored in the process of solving a problem. In this item, the process map can be useful to visualize the problem-solving process and play a complementary role in descriptive statistics. For example, if a student took a long time over a particular sequence related to problem-solving, this could be interpreted in two ways. One is the case in which many types of sequences are explored, and another is the case in which only a few sequences are explored but very carefully for a long time. In these cases, it is impossible to identify the problem-solving patterns of the students only by using descriptive statistics. However, the process map can provide information about the time spent on each step and the percentage of students who clicked a specific button at each step which would not have been elicited from descriptive statistics alone. Therefore, we adopted the process map for a more accurate and richer interpretation of the students’ problem-solving behavior analysis. In addition, when we clustered the students, we tried not to be limited to the boundaries of countries or cultures, but focus instead on students’ characteristics. After clustering, we identified those demographic factors such as which countries’ students mostly belong to each cluster. By doing this, we tried to capture the characteristics of countries with a low percentage of students with the correct answer, which had not been covered much so far. We used R software version 4.0.3 (R Core Team, 2020) for variable selection [glmnet package (Hastie and Qian, 2016)] and the visualizing process map [processmapR package (Janssenswillen et al., 2022)] and used Python software (version 3.10.2) for clustering.

### Item description and data processing

**Figure 1:**
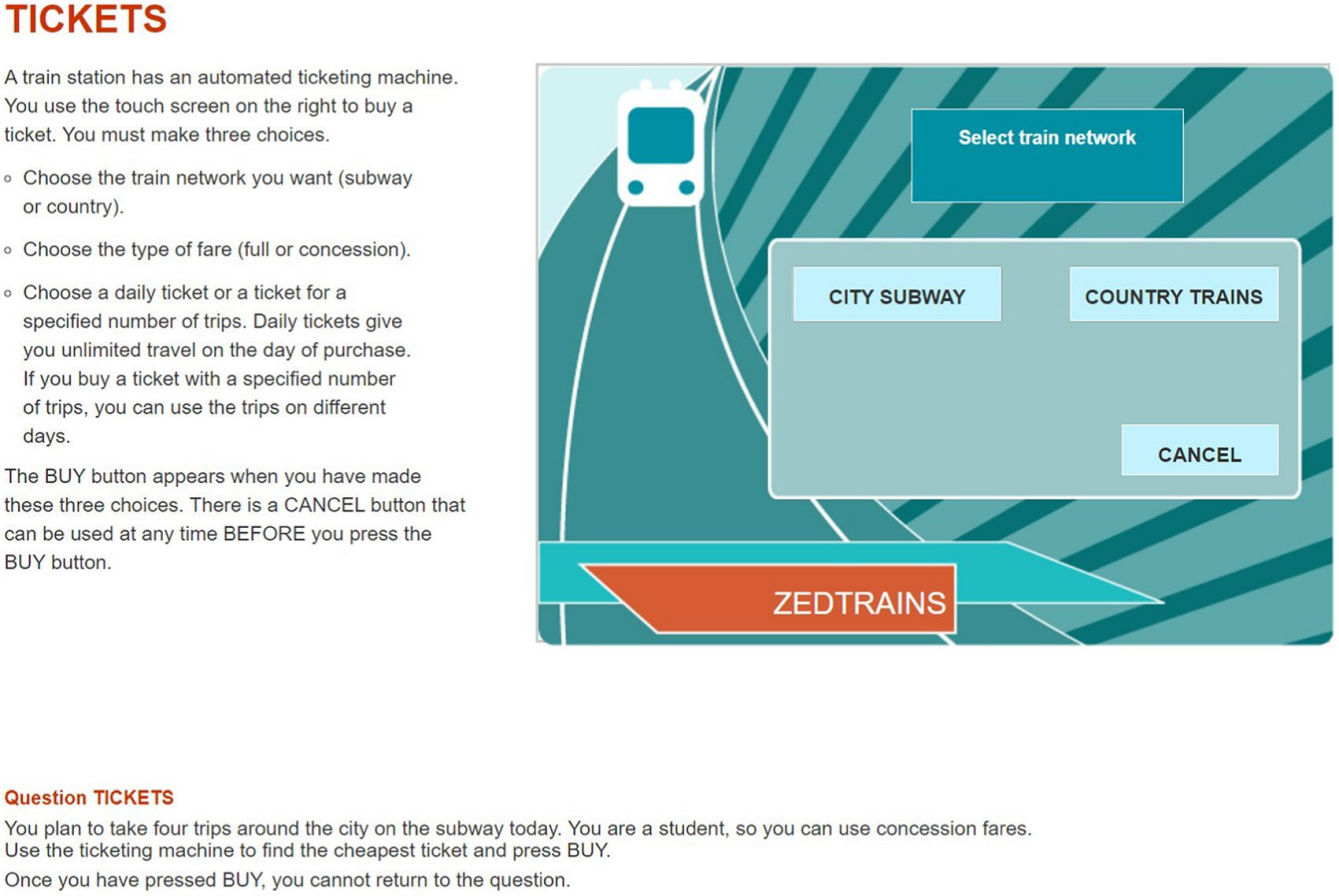
A snapshot of the first page of the problem- solving item (CP038q1) in Programme for International Student Assessment, 2012. Reproduced with permission from OECD, Tickets, PISA Test 2012 © OECD, 2012, https://www.oecd.org/pisa/test-2012/testquestions/question5/, Accessed on (05.04.22).

**Figure 2:**
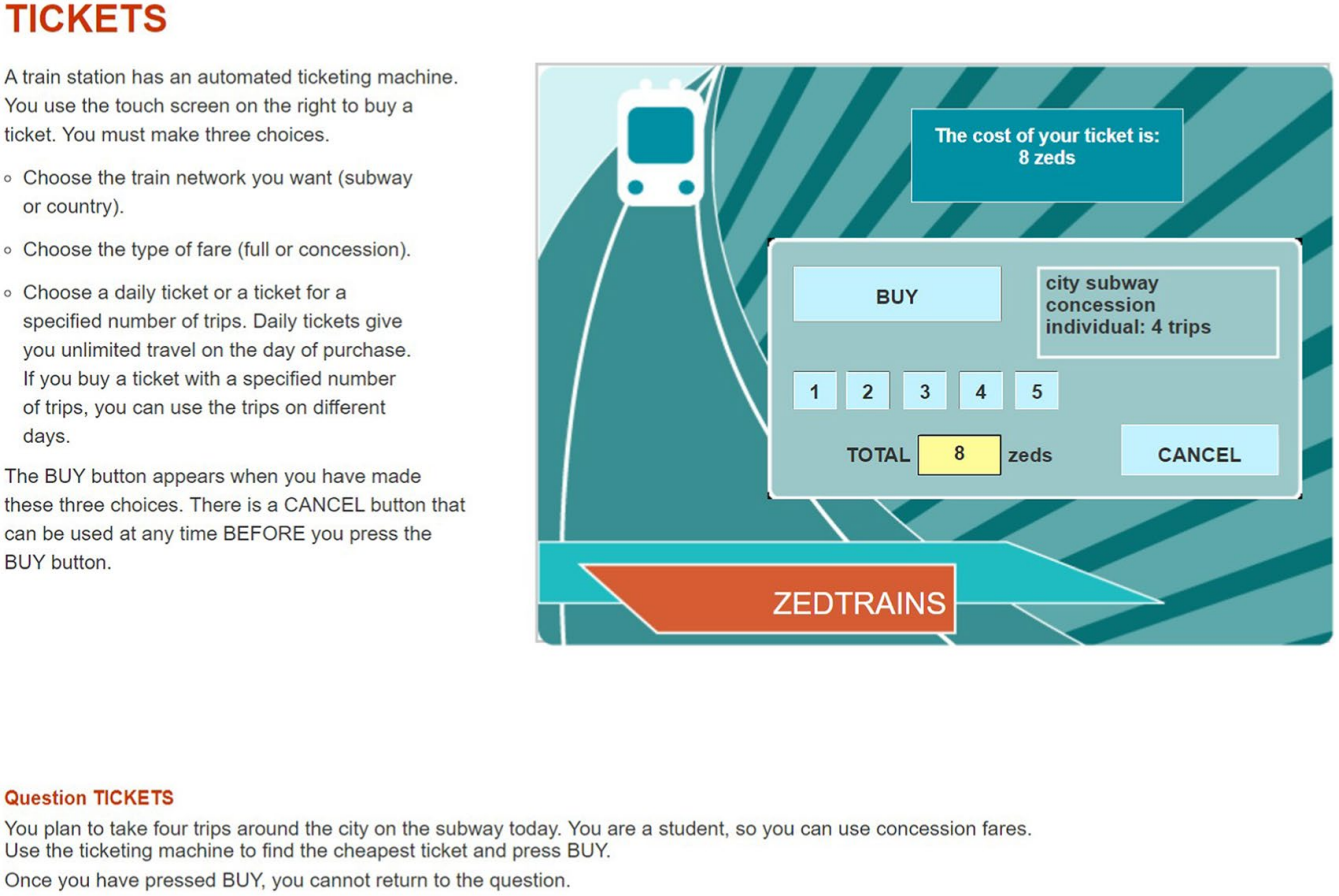
A snapshot of the last page of the problem- solving item (CP038q1) in Programme for International Student Assessment, 2012. Reproduced with permission from OECD, Tickets, PISA Test 2012 © OECD, 2012, https://www.oecd.org/pisa/test-2012/testquestions/question5/, Accessed on (05.04.22).

The item used in this study was CP038q01 in PISA 2012 CPS. The PISA 2012 CPS included 16 units with a total of 42 items. The OECD has provided sample items, such as a Vending Machine, a Vacuum Cleaner, and Climate Control. We decided to use one item from the CPS unit Vending Machine for our analysis. This is because it fits well with the theoretical concept of CPS and constitutes an appropriate selection, representing an important part of the CPS framework at PISA 2012. As shown in Figure 1, students are required to click the button on a ticket vending machine and buy the cheapest one offered among the tickets that satisfy the given conditions. The available buttons on the vending machine are, sequentially, “CITY SUBWAY” (hereinafter “CITY”) or “COUNTRY TRAINS” (hereinafter “COUNTRY”), then “FULL FARE” or “CONCESSION,” then “DAILY” or “INDIVIDUAL.” If the student clicks “INDIVIDUAL” in the third step, as illustrated in Figure 2, a screen shows the selected conditions of the ticket and asks for the number of tickets from 1 to 5. The final price depends on the number of tickets purchased. After determining the number of tickets, the student has the choice of clicking “BUY” to complete the item by buying the ticket(s) or clicking “CANCEL” to explore other tickets further. Meanwhile, if you click “DAILY” at the third step, the next screen shows the price of the ticket that satisfies the conditions selected so far, and the student is asked to choose whether to complete the item by clicking “BUY” or instead, to explore other tickets with different conditions by clicking “CANCEL.” Except for the button that determines the number of tickets, clicking any button in a specific step automatically advances one to the next step, and there is no way to go back to the previous step. The only way to go back to the previous step is to click “CANCEL,” which is in each step, and if “CANCEL” is clicked, the ticket conditions set so far will be reset and the student will be returned to the first step at the same time. Eight combinations of ticket conditions can be set in this item (excluding the ticket number condition), and the total combination of tickets including the number of tickets is 24. Two tickets satisfy the conditions given in the item: “CITY”–“CONCESSION”–“INDIVIDUAL”–“4 tickets” and “CITY”–“CONCESSION”–“DAILY.” To address this item, the student needs to compare the prices of the tickets with two conditions, the former ticket price of 8 Zed, and the latter is 9 Zed. Since the former ticket price is cheaper, the correct answer is to select “CITY”–“CONCESSION”–“INDIVIDUAL”–“4 tickets” and then “BUY”.

In PISA 2012, a partial scoring system—0 for incorrect, 1 for partially correct, and 2 for correct—is applied for some items, and the *Ticket* item is one of them. With this item, students who purchase the cheapest ticket after comparing the prices of two tickets that otherwise satisfy all the given conditions would then receive 2 points (full credit). Other students who only check one of the two tickets and purchase the ticket without comparison would receive 1 point (partial credit). All other cases are scored as 0 points (no credit). Of a total of 30,452 students who addressed this item, 7,195 got full credit. The subjects of this study are those students with the correct answer (i.e., the students who received full credit).^1^

In addition, in the case of PISA 2012 problem-solving, there was no limit on the time spent addressing each item; however, a 20-min limit was set for the whole test. Either one or two clusters were randomly assigned to students depending on different assessment designs (OECD, 2014a). For the *Ticket* item (CP038q1), the students’ average time spent was 54.8 s, and the median value was 50.2 s, while the average time spent by students with the correct answer was 67.3 s, and the median value was 62.2 s.

### Variable generation

In order to analyze the problem-solving patterns of the students with the correct answer, variables needed to be generated in light of the number of possible cases in the problem-solving process. Before generating variables, of the 7,195 students who received full credit in this item, 7,191 students remained, excluding four students who did not have a start (“START_ITEM”) or end (“END_ITEM”) of problem-solving in the raw data. After this, all actions of each student are sorted in chronological order. Regarding the number of tickets, 4 tickets are coded as “trip_4,” and all other buttons (1, 2, 3, 5) are coded as “trip_n.” This is because the number of tickets other than “trip_4” has the same importance in terms of correct-answer-related actions and strategies. By combining them into “trip_n,” we reduced the logical number of possible action sequences. Also, when “trip_n” is repeated consecutively or the same action is recorded several times in a row, only the first action is left (e.g., trip_n, trip_n, trip_n, trip_n → trip_n). Using clean data, two sequences that satisfy the condition given in the item are defined and divided into “a” and “b” (b1, b2, b3, b4), respectively, as shown in Table 1. Specifically, the “b” (b1 ~ b4) sequence, which is directly related to the correct answer, is divided according to whether the intended purpose is “decision-making” or “exploring various tickets,” and whether it includes only “trip_4” or both “trip_4” and “trip_n.” The number of possible cases is defined as “b1,” “b2,” “b3,” and “b4,” respectively. Among them, “b1” and “b2” are the sequences of “decision-making,” ending with “BUY.” They mean that the item is addressed by purchasing the ticket with the answer-related conditions. To be specific, “b1” is the most efficient sequence that selects “trip_4” without exploring any other combination of tickets, while “b2” includes both “trip_n” and “trip_4” but ends with “trip_4” and “BUY.”

**Table 1:** Lists of answer-related sequences.

Name	Sequences
a	city→concession→daily→cancel
b1	city→concession→individual→trip_4 → buy
b2	city→concession→individual→trip_n → trip_4 → buy
	city→concession→individual→trip_n → trip_4 → trip_n → trip_4 → buy
	city→concession→individual→trip_n → trip_4 → trip_n → trip_4 → trip_n → trip_4 → buy
	city→concession→individual→trip_n → trip_4 → trip_n → trip_4 → trip_n → trip_4 → trip_n → trip_4 → buy
	city→concession→individual→trip_4 → trip_n → trip_4 → buy
	city→concession→individual→trip_4 → trip_n → trip_4 → trip_n → trip_4 → buy
	city→concession→individual→trip_4 → trip_n → trip_4 → trip_n → trip_4 → trip_n → trip_4 → buy
	city→concession→individual→trip_4 → trip_n → trip_4 → trip_n → trip_4 → trip_n → trip_4 → trip_n → trip_4 → buy
b3	city→concession→individual→trip_4 → cancel
b4	city→concession→individual→trip_n → trip_4 → cancel
	city→concession→individual→trip_n → trip_4 → trip_n → cancel
	city→concession→individual→trip_n → trip_4 → trip_n → trip_4 → cancel
	city→concession→individual→trip_n → trip_4 → trip_n → trip_4 → trip_n → cancel
	city→concession→individual→trip_n → trip_4 → trip_n → trip_4 → trip_n → trip_4 → cancel
	city→concession→individual→trip_n → trip_4 → trip_n → trip_4 → trip_n → trip_4 → trip_n → cancel
	city→concession→individual→trip_n → trip_4 → trip_n → trip_4 → trip_n → trip_4 → trip_n → trip_4 → cancel
	city→concession→individual→trip_4 → trip_n → cancel
	city→concession→individual→trip_4 → trip_n → trip_4 → cancel
	city→concession→individual→trip_4 → trip_n → trip_4 → trip_n → cancel
	city→concession→individual→trip_4 → trip_n → trip_4 → trip_n → trip_4 → cancel
	city→concession→individual→trip_4 → trip_n → trip_4 → trip_n → trip_4 → trip_n → cancel
	city→concession→individual→trip_4 → trip_n → trip_4 → trip_n → trip_4 → trip_n → trip_4 → cancel
	city→concession→individual→trip_4 → trip_n → trip_4 → trip_n → trip_4 → trip_n → trip_4 → trip_n → cancel
	city→concession→individual→trip_4 → trip_n → trip_4 → trip_n → trip_4 → trip_n → trip_4 → trip_n → trip_4 → cancel

**Figure 3:**
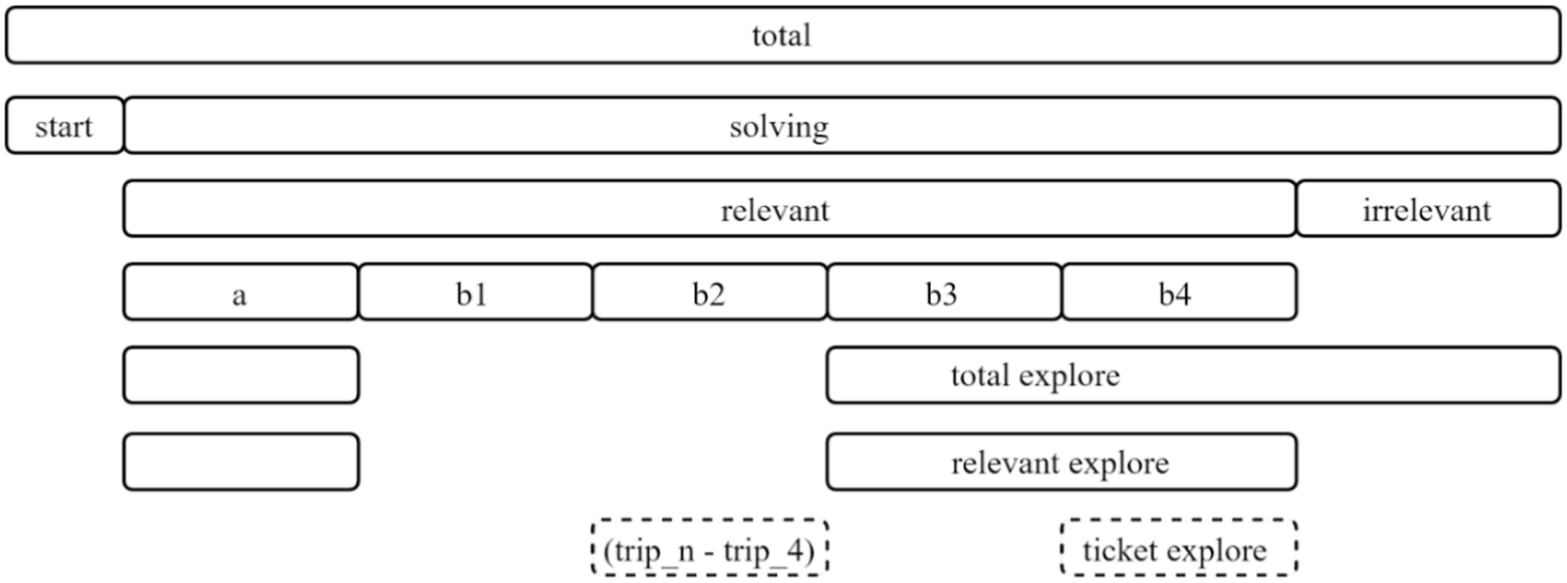
Structure of variables.

Meanwhile, “a,” “b3,” and “b4” are sequences that explore answer-related tickets but end with “CANCEL.” Among them, “b3” is the most efficient sequence where “CANCEL” is clicked to thereby explore other tickets after checking only the answer-related 4 tickets(“trip_4”). “b4” is the sequence that includes both “trip_4” and “trip_n” before clicking “CANCEL.” In the case of “b2” and “b4,” it is confirmed that the ticket number selection (“trip_4,” “trip_n”) is repeated a maximum of four times, i.e., only the sequences where “trip_n” and “trip_4” are repeated up to four times and are included in the clean data. After defining the sequences as shown in Table 1, various problem-solving steps that can be identified in the item are defined. Based on the steps, variables are generated as shown in Table 2. Figure 3 illustrates the relationships between the variables.

**Table 2:** List of variables and their explanations.

Variables	Variable explanation
time_start	Total time from presenting the item to the student until clicking the first button to address the problem. This denotes the time it took each student to understand the problem.
time_total	Total time from presenting the item to completion (when “BUY” was clicked).
time_a	Total time spent performing the “a” sequence (city → concession → daily → cancel). This is obtained by subtracting the timestamp when “CITY” was clicked from the timestamp when “CANCEL” was clicked in one “a” sequence.
time_b1	Total time spent performing the “b1” sequence (city → concession → individual → trip_4 → buy). This is obtained by subtracting the timestamp when “CITY” was clicked from the timestamp when “BUY” was clicked in a “b1” sequence. *This is the most efficient decision-making process possible in the whole problem-solving process.*
time_b2	Total time spent performing the “b2” sequence (city → concession → individual → trip_n/trip_4 → … → trip_4 → buy). This is obtained by subtracting the timestamp from when “CITY” was clicked from the timestamp to when “BUY” was clicked in a “b2” sequence. *The “b2” sequence is not the most efficient path, but it is a decision-making process related to the correct answer.*
time_b3	Total time spent performing the “b3” sequence (city → concession → individual → trip_4 → cancel). This is obtained by subtracting the timestamp from when “CITY” was clicked from the timestamp to when “CANCEL” was clicked in a “b3” sequence. *This is the most efficient exploring process possible in the problem-solving process.*
time_b4	Total time spent performing the “b4” sequence (city → concession → individual → trip_n/trip_4 → … → trip_n/trip_4 → cancel). This is obtained by subtracting the timestamp from when “CITY” was clicked from the timestamp to when “CANCEL” was clicked in a “b4” sequence. *This is not the most efficient, but it is an “exploring process” related to the correct answer.*
time_answer	Total time spent performing all “a” and “b” (b1, b2, b3, b4) sequences, which are related to the correct answer conditions. (time_a + time_b1 + time_b2 + time_b3 + time_b4)
time_irrelevant	Total time spent exploring sequences that are not related to the correct answer conditions. (time_total - time_start - time_answer)
time_solving	Total time each student spent clicking the button and taking actual actions to solve the problem. (time_total - time_start)
time_ticket_explore	Total time spent repeatedly exploring “trip_n” and “trip_4” in “b2” and “b4” sequences. Unlike other buttons, students can alter the number of tickets several times on the same screen without clicking “CANCEL.” We thought that there would be a difference in the strategy or problem-solving pattern between the students who immediately clicked “trip_4,” which is directly related to the correct answer, and the students who selected “trip_4” after navigating other numbers of tickets buttons.
time_explore_relevant	Total time spent exploring the answer-related sequences. This is because, while some students might strategically explore the correct answer-related sequence only once, others decide on the final answer after exploring other sequences several times. (time_a + time_b3 + time_b4)
time_explore_total	Total time spent exploring other sequences before clicking the first button of sequence “b1” or “b2,” which end with “BUY.” (time_total - (time_start+time_b1 + time_b2))
time_avg_explore	The average time spent exploring the correct answer-related sequences (a, b1, b2, b3, b4). (time_answer/length)
events_num	The total number of events recorded in log data after cleaning. This includes “START_ITEM” and “END_ITEM” which indicate when the item had been presented and completed.
time_avg_btw_events	The average time between button clicks. This denotes how much time each student spent thinking about and choosing the next step, and how carefully each student clicked the button. (time_solving/(event_num-3))
length	The total number of times that the correct answer-related sequence (a, b1, b2, b3, b4) was executed. (e.g., if “a,” “b3,” “b4,” and “b2” were performed, the length is 4.)

### Variable selection method

Among the generated variables, the variable selection method was used to select variables that are important to explain the problem-solving ability of students with the correct answer. Then, selected variables were used for clustering. The method used in this study was the penalized regression method, which is one of the widely used methods in variable selection (Yoo, 2018). Penalized regression methods, also called shrinkage methods, are methods of selecting variables by reducing regression coefficients using a penalty function (Tibshirani, 1996). By continuously penalizing coefficients with a regularization parameter, penalized regression methods are known to produce more stable models than discrete methods such as forward selection or backward elimination (Yoo, 2018). The most widely used penalized regression methods are Ridge (Hoerl and Kennard, 1970a), LASSO (Least Absolute Shrinkage and Selection Operator, Tibshirani, 1996), and Elastic Net (Zou and Hastie, 2005). In this study, Elastic Net is applied, taking into account the characteristics of the generated time-related variables.

Elastic Net is a regularization and variable selection technique developed by Zou and Hastie (2005). Elastic Net uses both *L*1 and *L*2 penalties by combining Ridge (Hoerl and Kennard, 1970b) with LASSO, and has the advantage of being able to address both variable selection, which is the strength of LASSO, and multicollinearity, which is the strength of Ridge. Specifically, it is characterized by better performance than variable selection and LASSO on collinear data (Zou and Hastie, 2005). Time-related variables are generated by dividing the total time according to the detailed steps of problem-solving or by combining steps with similar characteristics. Due to the nature of time-related variables, the multicollinearity problem inevitably arises. Since all generated variables have their meaning in terms of addressing this item, we try to work out this problem not by dropping certain problematic variables arbitrarily but by using a statistical method. Considering all, Elastic Net is the most suitable statistical method for selecting the variables. The equation for estimating the regression coefficients of Elastic Net is as follows:


$$\hat{\beta }=\underset{\beta }{\arg\min}\left\{\frac{1}{2}\overset{N}{\underset{i=1}{\sum }}{\left(y_{i}-\beta_{0}-\overset{P}{\underset{j=1}{\sum }}x_{ij}\beta_{j}\right)}^{2}+\lambda \overset{P}{\underset{j=1}{\sum }}\beta_{j}^{2}\right\}$$

In the above equation, $\hat{\beta }$ indicates the vector of shrunk coefficients of *j* predictors. The second term on the right side is a penalty function, which is a combination of the *L*1 norm and the *L*2 norm. Elastic Net has two tuning parameters, α and λ. First, λ is a regularization (or penalty) parameter, which controls the extent of regularization as in LASSO. The larger λ means that the coefficient shrinks closer to zero and the smaller λ means the coefficient is closer to the least square estimation (Yoo, 2018). Next, α is a tuning parameter that connects Ridge and LASSO. If the value of α approaches 1, it approximates to LASSO, and if it approaches 0, it approximates to the Ridge. In general, it is considered relatively more important to determine the degree of regulation between the two tuning parameters, so it is not necessary to justify both tuning parameters (Yoo, 2021). Therefore, it is common to select the value of α at the researcher’s discretion (T. Hastie, personal communication, February 9, 2017, as cited in Yoo, 2021).

## Results

### RQ1: Variable selection results

We used Elastic Net to select the most important variables to explain the overall PISA 2012 problem-solving ability of those students with the correct answer. For the analysis, we divided all the data randomly into a test set and a training set, by dividing the data by 7 to 3. Then, after fitting the model with 10-fold cross-validation on the training set, we obtained a prediction error with the test set. This selection process was repeated 100 times, and we used the variables which are selected 100 times out of a total of 100 times as criteria for clustering.

We set the dependent variable as the overall problem-solving ability in PISA 2012, which is calculated as the ratio of the student’s total score (the sum of credits they received) and the possible maximum score that the student could receive in the corresponding booklet. This is because, since PISA 2012 depends on matrix sampling, the booklet presented to each country or student is different, and the number of items, their difficulty, and the maximum score of each booklet are also different. Thus, it is inappropriate to use the ratio of the simply added-up score for the dependent variable. As an alternative, we used the PISA scale score as reported in the PISA 2012 results (OECD, 2014b), which was scaled with a mean of 500 and a standard deviation of 100, considering the characteristics of each item. For all CPS items in the booklet, the threshold of the PISA scale score was assigned according to the actual credits (0, 1, and 2) that each student received from each item. By using the ratio of the PISA scale score as the dependent variable, the possible differences in scores between students who received different booklets were compensated for and students’ overall problem-solving ability in PISA 2012 could be accurately reflected simultaneously.

**Figure 4:**
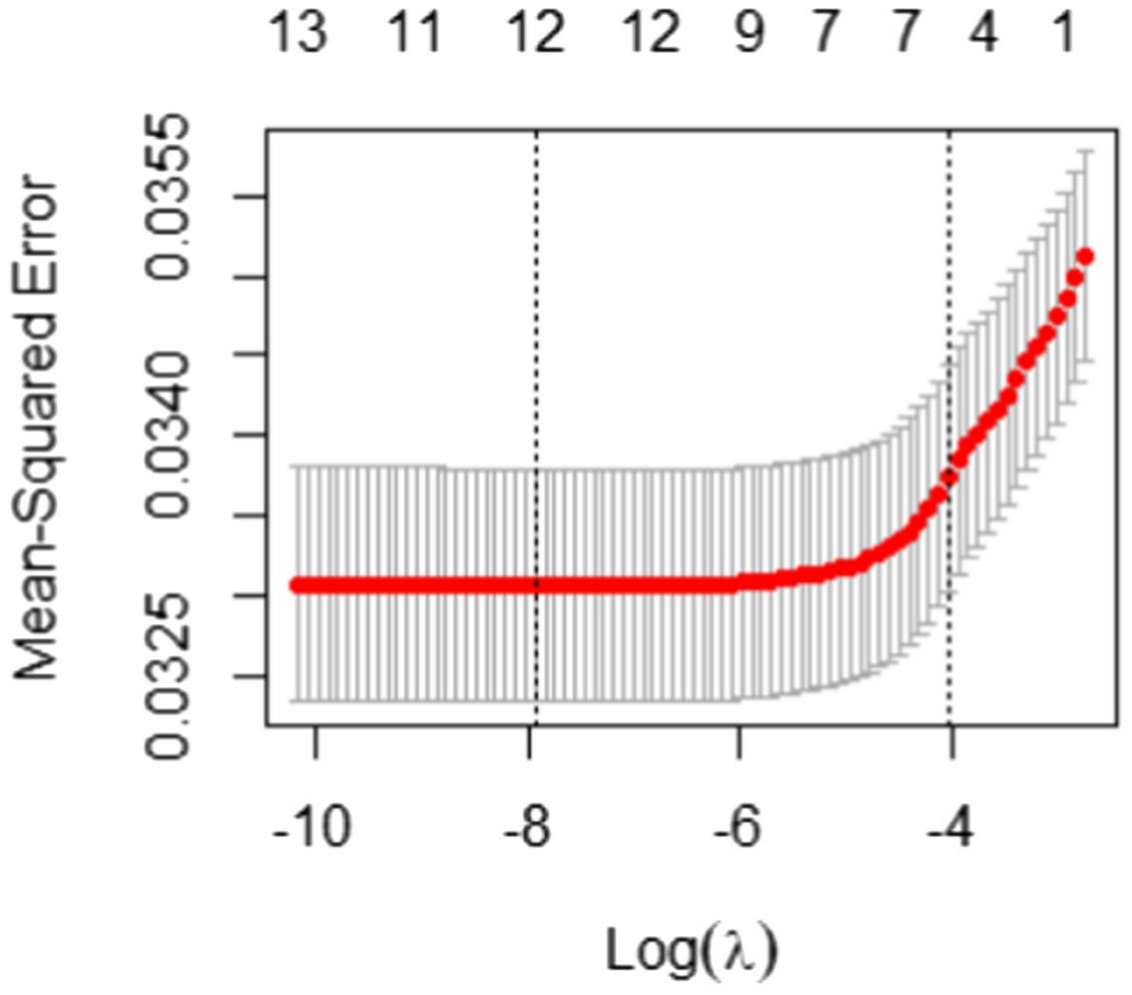
10-fold cross-validation result with mean-squared error for students with the correct answer.

In Elastic Net, as suggested by Hastie and Qian (2016), we set α to 0.5 to take advantage of both Ridge and LASSO equally. Another tuning parameter λ was taken through the 10-fold cross-validation and Figure 4 illustrates the result of the 10-fold cross-validation with the Mean-Squared Error (MSE). The vertical dotted lines in Figure 4 are the upper and lower bounds of the one-standard-error rule. In the plot, the number of non-zero coefficients with the upper bound corresponds to 5. With the results of variable selection counts (Table 3), we selected a total of five variables for the most parsimonious model.

**Table 3:** Variables selection counts out of 100-times repeats for students with the correct answer.

Variable	Selection counts
time_a	47
time_b1	100
time_b2	90
time_b3	100
time_b4	75
time_start	100
time_solving	1
time_total	8
time_answer	19
time_irrelevant	100
time_explore_relevant	1
time_ticket_explore	83
time_explore_total	1
time_avg_explore	89
tme_avg_btw_events	100
events_num	73
length	90

As shown in Table 3, “time_b1,” “time_b3,” “time_start,” “time_irrelevant,” and “time_avg_btw_events” were selected through Elastic Net. The test set has a good RMSE of 0.18.

### RQ2: Problem-solving patterns by clusters

**Figure 5:**
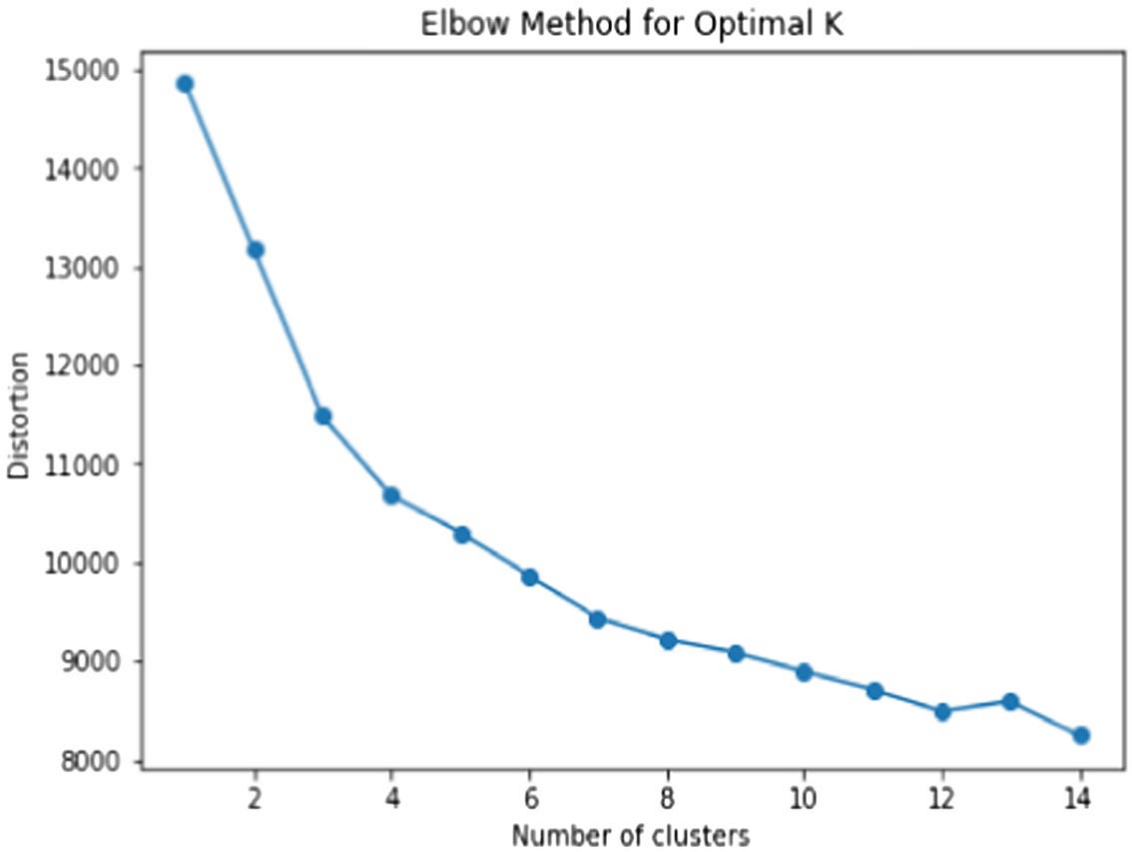
Result of elbow method for determining optimal *k* for students with the correct answer.

Students were clustered using the *k*-medoids method using the five previously selected variables. The *k*-medoids method uses minimal dissimilarity to all objects in a cluster as the determinant that is opposite to the distance in the *k*-means method. Before clustering, we determined the optimal number of clusters using the elbow method. The result is shown in Figure 5. In the graph, the elbow point is *k* = 4, and we clustered the students into four groups.

**Figure 6:**
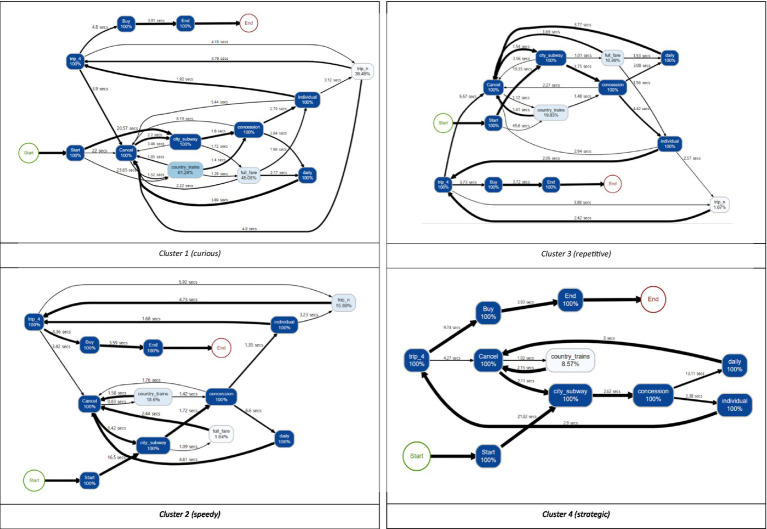
Process maps for four clusters for students with the correct answer.

Table 4; Figure 6 show the results of the descriptive statistics and process map of each cluster, respectively. Specifically, the process map is filtered by 0.8 based on the students’ trace frequency. In the process map, the number written on the edge shows the average time that students took to explore the path. The thickness of the edge indicates the percentage of the frequency of passing the path. In addition, each step to buying a ticket in the item is expressed as a node, and the number in the node is the percentage of students who clicked the node.

**Table 4:** Descriptive statistics (mean (standard deviation)) of variables by cluster.

Variable	Cluster 1 (curious)	Cluster 2 (speedy)	Cluster 3 (repetitive)	Cluster 4 (strategic)
	(*n* = 823)	(*n* = 2,412)	(*n* = 2,285)	(*n* = 1,671)
time_start*	20.90 s (12.88)	16.51 s (7.28)	19.28 s (10.01)	22.48 s (12.89)
time_b1*	8.47 s (6.31)	6.89 s (3.46)	7.21 s (3.37)	16.25 s (7.35)
time_b3*	7.22 s (9.63)	1.28 s (2.80)	19.00s (10.26)	1.24 s (4.63)
time_irrelevant*	31.68 s (17.27)	3.60s (3.10)	6.13 s (3.85)	3.33 s (3.42)
time_avg_btw_events*	3.16 s (1.72)	2.87 s (0.98)	2.72 s (0.96)	5.22 s (1.88)
time_a	16.09 s (14.48)	15.94 s (8.62)	11.75 s (10.25)	25.26 s (14.51)
time_b2	1.63 s (6.62)	2.17 s (6.40)	0.40s (3.57)	0.54 s (4.49)
time_b4	2.55 s (8.18)	2.65 s (8.13)	0.287 s (2.69)	0.93 s (6.91)
time_solving	67.64 s (27.61)	32.53 s (13.19)	44.78 s (15.07)	47.57 s (17.79)
time_total	88.53 s (32.91)	49.04 s (14.86)	64.07 s (18.60)	70.05 s (22.21)
time_answer	35.96 s (19.05)	28.93 s (12.06)	38.65 s (14.49)	44.23 s (16.72)
time_explore_relevant	25.86 s (18.16)	19.87 s (11.51)	31.04 s (13.72)	27.43 s (15.52)
time_ticket_explore	1.51 s (4.35)	1.68 s (4.14)	0.21 s (1.46)	0.42 s (3.02)
time_explore_total	60.81 s (28.50)	27.82 s (17.91)	37.98 s (16.13)	31.85 s (19.12)
time_avg_explore	11.24 s (6.48)	11.62 s (4.60)	11.07 s (4.19)	20.37 s (7.49)
events_num	24.61 (11.05)	11.95 (4.75)	17.07 (4.67)	9.41 (2.96)
length	3.52 (1.64)	2.65 (1.13)	3.61 (1.05)	2.24 (0.74)

*Variables used for clustering.

Cluster 1 (curious) had the smallest number of students (823) among the four clusters. This cluster characterized the long “time_irrelevant,” which means that sequences of the actions other than the correct answer-related ones were explored in various ways. It can also be seen from the process map that Cluster 1 is close to a spaghetti shape, that is, it is an entangled and complex problem-solving process. In the process map, the percentage of clicking buttons that do not correspond to the correct answer conditions (e.g., “COUNTRY,” “FULL FARE,” “trip_n”) was higher than in other clusters, which is related to the longest value of “time_irrelevant.” In addition, “length,” “time_explore_total,” and “event_num” were also long. Based on all information, Cluster 1 explored many other sequences as well as those sequences related to the correct answer. Since students in this cluster are learning through trial and error, it is necessary to know that exploring only the correct-answer-related things is not always the optimal way. Also, a teacher needs to respect their various interests and present abundant learning materials which may help draw their attention.

Cluster 2 (speedy) had the largest number of students (2,412) among the four clusters. In this cluster, the values of all variables used for clustering were relatively small compared to other clusters. “time_start,” especially, had the smallest value across the clusters, and “time_b3” and “time_irrelevant” also had small values. With a process map of Cluster 2, the map shows that students mainly explored those sequences related to the correct answer. Thus, the students did everything quickly, including understanding the given conditions, setting up a problem-solving strategy, and exploring the strategy. Also, the short “time_avg_btw_events” implies that the decisions were made over a short time when deciding which button to click in each step. When exploring the tickets, even though some students made some mistakes, such as clicking “COUNTRY,” which is not related to the correct answer, they quickly realized them and tried to make things right by clicking “CANCEL” without going any further. For the students in this cluster, it could be helpful to let them know that they made mistakes quite frequently during the problem-solving process and they did not need to hurry.

Cluster 3 (repetitive) is characterized by exploring the correct answer repetitively compared to other clusters. This is because “time_a,” “time_b2,” and “time_b4” are short while “length” is long. Based on this, it can be inferred that “time_b3” has a large value not because the sequence of “CITY-CONCESSION-INDIVIDUAL-trip_4-CANCEL” was explored for a long time, but because the sequence had been explored repeatedly several times. Furthermore, in the process map, a relatively large percentage of students clicked buttons that did not meet the conditions in the item such as “FULL FARE” or “COUNTRY.” Also, they did not click “CANCEL” immediately when they clicked the wrong button. Instead, they tended to keep exploring the sequence such as “CONCESSION” after “COUNTRY.” In summary, Cluster 3 can be interpreted as that after exploring various sequences, the sequences corresponding to the correct answer were checked repeatedly to ensure correct decision-making. Given that the students did not correct the answer immediately, they need to practice thinking reflectively to make fewer mistakes, particularly on the test with time limits.

Cluster 4 (strategic) is characterized by fewer clicks and mistakes, but longer resolution times. As the process map shows, Cluster 4 spent a great deal of time addressing the problem and devising a strategy to solve it and spent a relatively long time clicking the button (“time_avg_btw_events”), carefully addressing the problem. In addition, this cluster did not explore some actions unnecessarily, such as “COUNTRY” and “trip_n.” Also, “time_b3” and “time_irrelevant” are short, so we can infer that most of the students solved this problem with the shortest correct-answer-related sequences, which are “a-b1” and “b3-a-b1.” The evidence can also be found in the process map that the edges from node “CONCESSION” are divided into two that have a similar thickness. In brief, they are students who solve the problem with one careful search based on the optimal strategy, rather than repeatedly searching the sequences to check whether their answer is correct. Considering that they are the students who think more and act less, so even though they do not react immediately, we need to acknowledge their style and wait for them.

### RQ3: Differences between problem-solving patterns of students with the correct answer and students with incorrect answers

**Figure 7:**
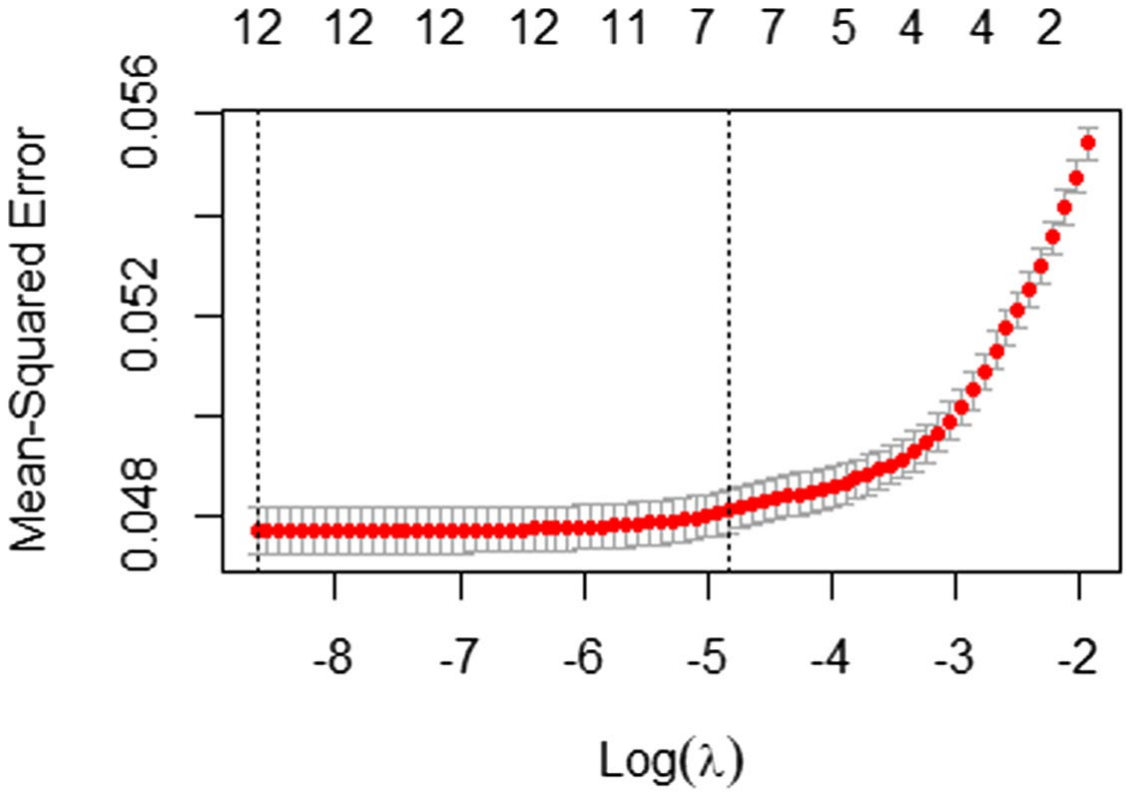
10-fold cross-validation result with mean-squared error for students with incorrect answers.

In order to verify the internal validity of this study, we also analyzed students with incorrect answers through the process given above. Through the Elastic Net, seven variables were selected (see Figure 7; Table 5). As shown in Table 5, “time_b1,” “time_b4,” “time_start,” “time_irrelevant,” “time_avg_btw_events,” “length,” and “events_num” were selected and the test set has a good RMSE of 0.21.

**Table 5:** Variables selection counts out of 100-times repeats for students with incorrect answers.

Variable	Selection counts
time_a	11
time_b1	91
time_b2	18
time_b3	1
time_b4	100
time_start	90
time_solving	3
time_total	30
time_answer	11
time_irrelevant	84
time_explore_relevant	10
time_ticket_explore	8
time_explore_total	10
time_avg_explore	10
tme_avg_btw_events	100
events_num	100
length	100

**Figure 8:**
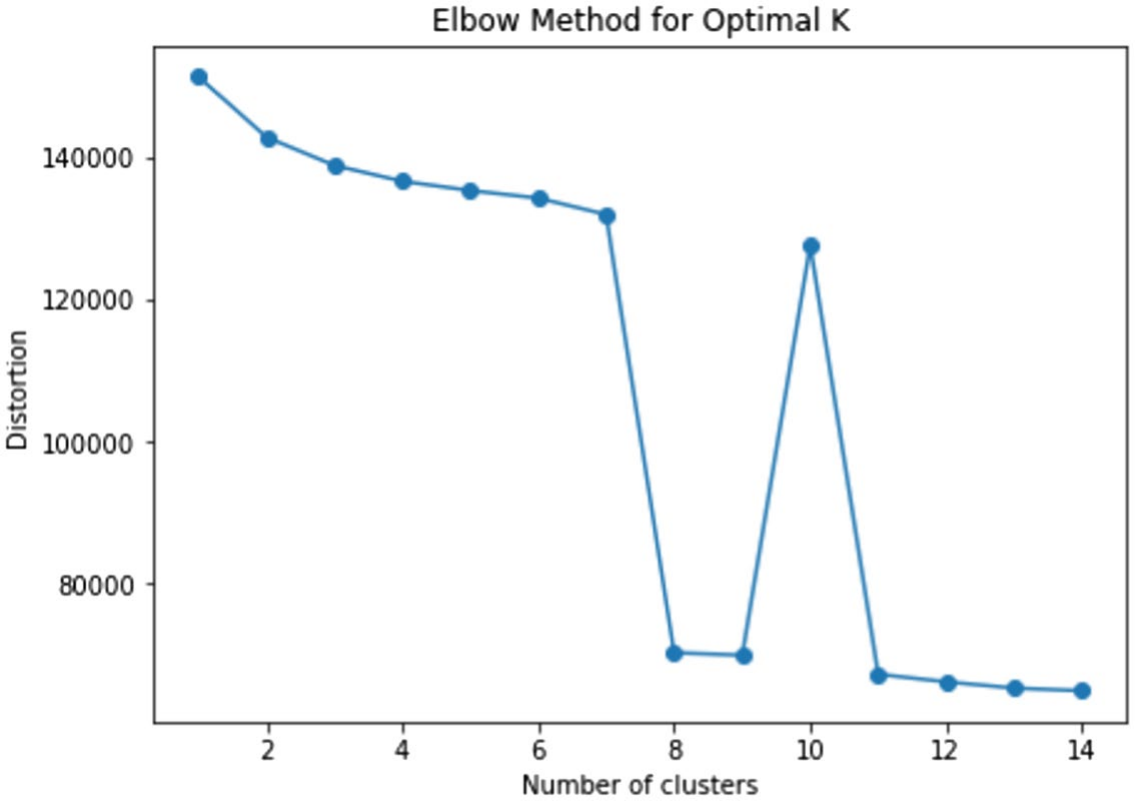
Result of elbow method for determining optimal *k* for students with incorrect answers.

**Figure 9:**
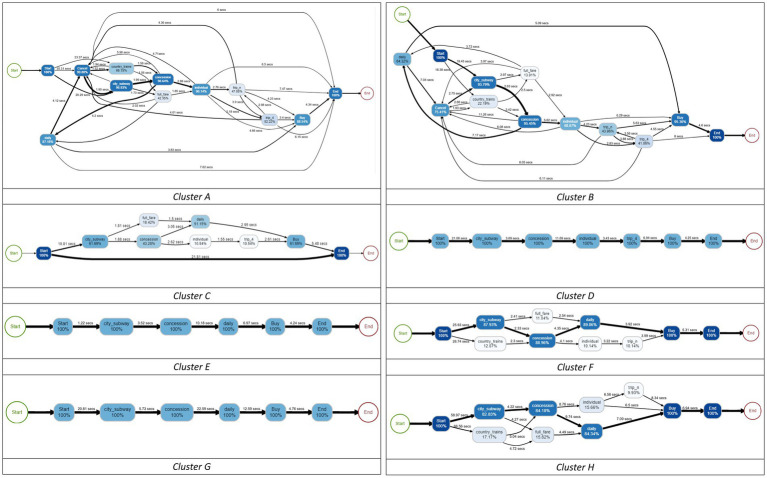
Process maps for eight clusters for students with incorrect answers.

Using these variables, the students were clustered into eight categories (see Figure 8), and the process map of each cluster is illustrated in Figure 9. Based on the four problem-solving patterns of students with the correct answer in RQ2, we were able to identify that these eight clusters can be classified similarly. In particular, Clusters A and B were similar to Custer 1 (curious); Cluster C was similar to Cluster 2 (speedy); and Clusters D–H were similar to Cluster 4 (strategic).

Unlike other clusters, Cluster A (*n* = 2,194) and Cluster B (*n* = 2,616) were the only clusters that clicked “CANCEL,” which means the students explored more than one path. That is the reason why these clusters showed more complex problem-solving processes than other clusters. Cluster A is more similar to Cluster 1 than Cluster B, in that students in this cluster explored irrelevant sequences, such as “COUNTRY” and “FULL FARE.”

Cluster C (*n* = 4,661) was similar to Cluster 2 in that all decisions were made over a relatively short period concerning other clusters. One of the idiosyncrasies is that the cluster includes students with the sequence of “START_ITEM →END_ITEM.” According to Zoanetti (2010), these students can be considered as they were not able to understand what to do.

Cluster D (*n* = 3,779), E (*n* = 3,345), F (*n* = 3,392), G (*n* = 1,918), and H (*n* = 1,344) can be interpreted as we interpreted Cluster 4 in that they had their problem-solving strategy, even though they partially understood the question of the item. They did not recognize that comparing two possible types of tickets was the intention of the item. Most of the students in Clusters E–H only explored sequence a*(city→concession→daily→buy), whereas Cluster D only explored b*(city→concession→individual→trip_n/trip_4 → buy). Nevertheless, in terms of the allocated time between the clusters, there were some differences. For example, Cluster E had relatively shorter “time_start” and longer “time_avg_btw_events,” whereas Cluster F showed the opposite patterns. In addition, Cluster G showed the longest “time_avg_btw_events,” and Cluster H showed the longest “time_start.”

## Summary and discussion

This study identified various problem-solving patterns of students found with the correct answer and compared them with the problem-solving patterns of students with incorrect answers. We captured students’ problem-solving characteristics based on their response time by directly accessing their process data of the PISA 2012 *Ticket* item (CP038q1) instead of using traditional methods such as self-reporting questionnaires. Specifically, we defined the crucial and necessary steps to address this item and generated variables with the time spent on each step and the number of actions in the problem-solving process. Then, we selected important variables for addressing the students’ overall problem-solving ability. Since generated time-related variables are derived from the total time and consist of the time spent on each step, they inevitably introduced multicollinearity problems. Elastic Net is a combination of LASSO (which can select variables) and Ridge (which can mitigate collinearity problems), so we adopted Elastic Net as the variable selection method.

Next, we categorized the students into four clusters using the *k*-medoids method with the selected variables. The results of analyzing and comparing characteristics between clusters based on descriptive statistics and process maps are as follows. First, Cluster 1 (curious) explored sequences unrelated to the correct answer in the most diverse way compared to other clusters. Second, Cluster 2 (speedy) spent the shortest time exploring the sequences and choosing the final answer. They did everything very quickly. Third, Cluster 3 (repetitive) chose the final answer after checking their answer repeatedly, though they made some mistakes exploring the correct-answer-related sequences. Lastly, Cluster 4 (strategic) spent a relatively long time reading the question phrasing of the item and devising an optimal solution strategy. Rather than checking the answer repeatedly, students in this cluster chose the answer after one careful search. As demonstrated, the four clusters were distinct. Given each cluster’s characteristics, feedback based on their problem-solving style is needed to improve students’ performance.

Finally, this study confirmed the internal validity of this approach by identifying the problem-solving patterns of students with incorrect answers. They were grouped into eight clusters, but considering their essential differences, they can be roughly classified into four clusters, making a pattern with a closer resemblance to the problem-solving patterns of students with the correct answer. Admittedly, given that they were students with incorrect answers, their problem-solving processes were more divergent.

Indeed, it cannot be said that one pattern is better than others. Each pattern has its way of approaching the item, and students can have the best strategy for their style as well as the characteristics of given items (Naumann, 2019). The fact that the problem-solving patterns were related to the item characteristics is also supported by the results of this study that the problem-solving patterns of students with incorrect answers can be also roughly classified similarly to the patterns of the students with the correct answer. In the same context, the values for each time-related variable should also be interpreted concerning individual abilities and item characteristics.

The study’s findings have important implications for researchers and educators. First, we accessed students’ actual problem-solving behavior directly through the process data, which were generated from the interactions between computers and humans. Unlike the self-reporting questionnaire, which could reflect examinees’ subjectivity, process data only include honest information about their problem-solving behaviors. Therefore, our results using process data revealed students’ problem-solving patterns objectively. Furthermore, we adopted the process map technique and visualized students’ cognitive processes that occurred during problem-solving. The process map is useful because it can not only show students’ problem-solving patterns (Figure 6) but also diagnose a student’s problem-solving behavior once their data are mapped. While previous studies were focused on short sequence units (e.g., Han et al., 2019; Ren et al., 2019), this study addressed problem-solving patterns using allotted time at the sequence-level while also aggregating them into process-level using a process map.

Second, we suggest that it is necessary to study the perspectives and positions of students who answered correctly, which have been difficult to investigate so far because they provide a single, identical correct answer. Indeed, this study reveals that the students show distinctly different characteristics when addressing a given problem. It is important to comprehend their diverse problem-solving patterns to devise learner-centered instructional designs and fulfill adaptive learning—such as providing appropriate feedback—for their further performance.

However, in terms of generalizability, this study has some limitations. Since this study is to showcase the potential of analyzing log data to identify problem-solving patterns of students, we analyzed one item as an example. Thus, to have external validity, the method that we proposed needs to be scrutinized using other items.

## Data availability statement

Publicly available datasets were analyzed in this study. This data can be found at: https://www.oecd.org/pisa/pisaproducts/database-cbapisa2012.htm.

## Ethics statement

Ethical review and approval was not required for the study on human participants in accordance with the local legislation and institutional requirements. Written informed consent from the participants’ legal guardian/next of kin was not required to participate in this study in accordance with the national legislation and the institutional requirements.

## Author contributions

H-JP, DL, and HP contributed to the study’s conception and design. H-JP was in charge of the paper administration, supervision, and validation of the paper. DL and HP wrote the first draft, data curation, and formal analysis. All authors contributed to the article and approved the submitted version.

## Conflict of interest

The authors declare that the research was conducted in the absence of any commercial or financial relationships that could be construed as a potential conflict of interest.

## Publisher’s note

All claims expressed in this article are solely those of the authors and do not necessarily represent those of their affiliated organizations, or those of the publisher, the editors and the reviewers. Any product that may be evaluated in this article, or claim that may be made by its manufacturer, is not guaranteed or endorsed by the publisher.

## Footnotes

^1^According to the PISA 2012 result (OECD, 2014a), students with partial credit correspond to Level 2, and they can test a simple hypothesis that is given to them and can solve a problem that has a single, specific constraint. While students who received full credit correspond to Level 5 on the problem-solving scale, they can systematically explore a complex problem scenario to gain an understanding of how relevant information is structured.
