# Corrigendum: Understanding students' problem-solving patterns: evidence from an allotted response time in a PISA 2012 item

**DOI:** 10.3389/fpsyg.2023.1138836

**Published:** 2023-05-10

**Authors:** Hyun-Jeong Park, Dayeon Lee, Hyemin Park

**Affiliations:** Department of Education, Seoul National University, Seoul, Republic of Korea

**Keywords:** process data, response time analysis, process map, learning process, problem-solving patterns, PISA 2012

In the published article, there was an error in Figure 9 as published. Figure 9 only displayed the panels for Clusters A–D. The panels for “Cluster E,” “Cluster F,” “Cluster G,” and “Cluster H” were missing. The corrected Figure 9 and its caption appear below.

**Figure 9:**
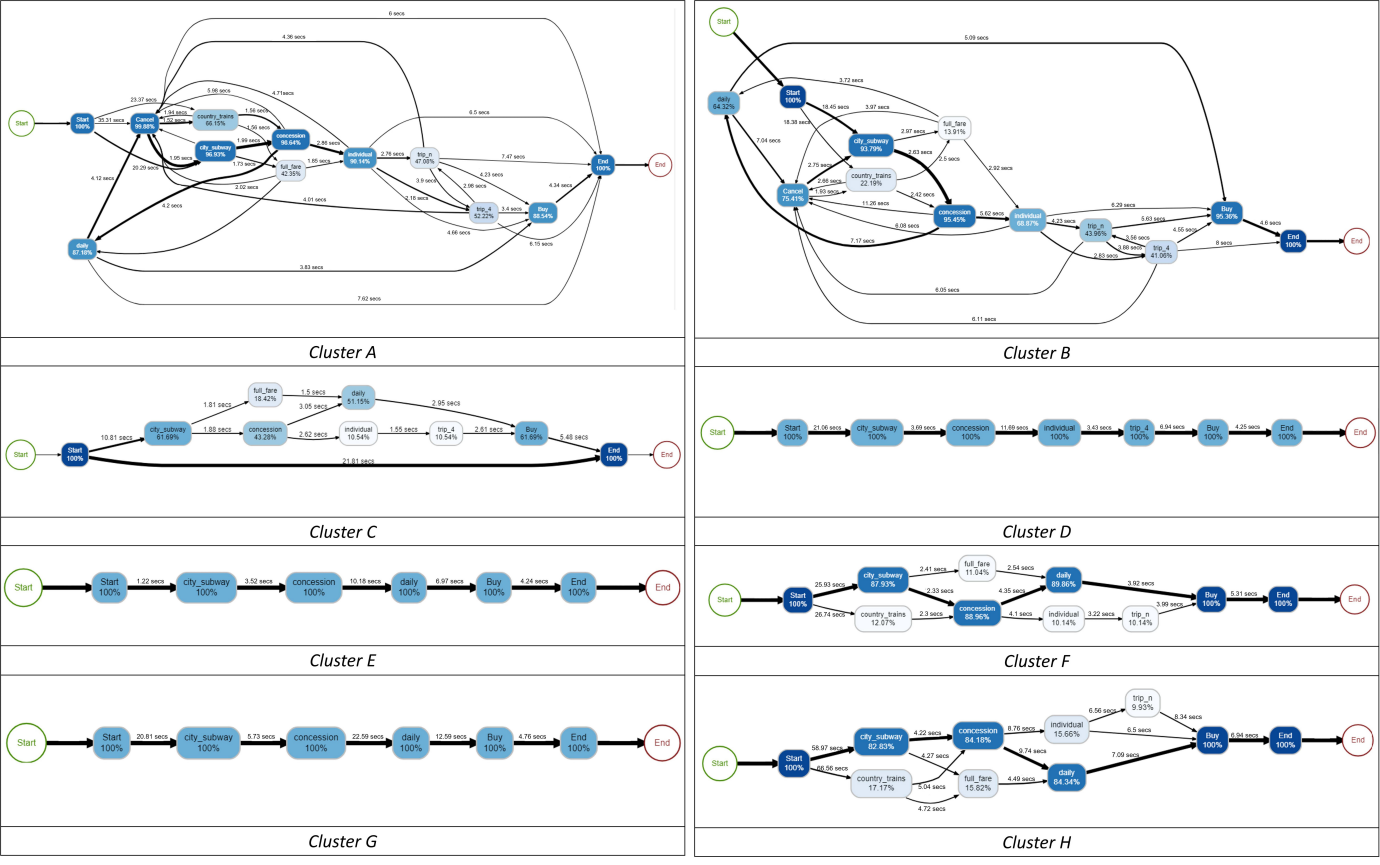
Process maps for eight clusters for students with incorrect answers.

The authors apologize for this error and state that this does not change the scientific conclusions of the article in any way. The original article has been updated.

